# A novel risk model based on the correlation between the expression of basement membrane genes and immune infiltration to predict the invasiveness of pituitary adenomas

**DOI:** 10.3389/fendo.2022.1079777

**Published:** 2023-01-04

**Authors:** Zheng Chen, Xin Sun, Yin Kang, Jian Zhang, Fang Jia, Xiyao Liu, Hongwei Zhu

**Affiliations:** ^1^ Department of Neurosurgery, The First Affiliated Hospital of Xiamen University, School of Medicine, Xiamen University, Xiamen, China; ^2^ Department of Immunology, Hokkaido University Graduate School of Medicine, Sapporo, Japan

**Keywords:** invasive pituitary adenomas, basement membrane genes, bioinformatics, PPI, GEO, tumor microenvironment, immune infiltration

## Abstract

**Objective:**

Invasive pituitary adenomas (IPAs) are common tumors of the nervous system tumors for which invasive growth can lead to difficult total resection and a high recurrence rate. The basement membrane (BM) is a special type of extracellular matrix and plays an important role in the invasion of pituitary adenomas (PAs). The aim of this study was to develop a risk model for predicting the invasiveness of PAs by analyzing the correlation between the expression of BM genes and immune infiltration.

**Methods:**

Four datasets, featuring samples IPAs and non-invasive pituitary adenomas (NIPAs), were obtained from the Gene Expression Omnibus database (GEO). R software was then used to identify differentially expressed genes (DEGs) and analyze their functional enrichment. Protein-protein interaction (PPI) network was used to screen BM genes, which were analyzed for immune infiltration; this led to the generation of a risk model based on the correlation between the expression of BM genes and immunity. A calibration curve and receiver operating characteristic (ROC) curve were used to evaluate and validate the model. Subsequently, the differential expression levels of BM genes between IPA and NIPA samples collected in surgery were verified by Quantitative Polymerase Chain Reaction (qPCR) and the prediction model was further evaluated. Finally, based on our analysis, we recommend potential drug targets for the treatment of IPAs.

**Results:**

The merged dataset identified 248 DEGs that were mainly enriching in signal transduction, the extracellular matrix and channel activity. The PPI network identified 11 BM genes from the DEGs: *SPARCL1, GPC3, LAMA1, SDC4, GPC4, ADAMTS8, LAMA2, LAMC3, SMOC1, LUM* and *THBS2*. Based on the complex correlation between these 11 genes and immune infiltration, a risk model was established to predict PAs invasiveness. Calibration curve and ROC curve analysis (area under the curve [AUC]: 0.7886194) confirmed the good predictive ability of the model. The consistency between the qPCR results and the bioinformatics results confirmed the reliability of data mining.

**Conclusion:**

Using a variety of bioinformatics methods, we developed a novel risk model to predict the probability of PAs invasion based on the correlation between 11 BM genes and immune infiltration. These findings may facilitate closer surveillance and early diagnosis to prevent or treat IPAs in patients and improve the clinical awareness of patients at high risk of IPAs.

## Introduction

Pituitary adenomas (PAs) are common neuroendocrine tumors that account for 10–15% of all primary tumors of the central nervous system (1). Most PAs are non-invasive, follow a slow growth pattern and remain in the intra sella (2). However, 25–55% of PAs may exhibit invasive features (Knosp grade III and IV), including invasion of the cavernous and sphenoidal sinuses and local or extensive bone erosion; these tumors are considered as invasive pituitary adenomas (IPAs) (3). Compared with non-invasive pituitary adenomas (NIPAs), IPAs are characterized by large volume, rapid proliferation, a high recurrence rate and a poor prognosis; moreover, these tumors are difficult to completely remove by surgery. Collectively, these factors can lead to serious damage being incurred by adjacent structures (3, 4). Despite numerous studies and advances in classification and prognosis, there are still no pathological marker that can be used to reliably predict the behavior of IPAs (5). Therefore, it is vital that we identify early diagnostic biomarkers for the clinicopathological behavior of IPAs and investigate the molecular mechanisms underlying the invasiveness of this condition.

An increasing number of studies have shown that the tumor microenvironment (TME) plays a crucial role in tumor progression and treatment (6). Therapeutic strategies targeting the TME have become a promising approach for the treatment of tumors (7). The TME consists of various types of immune cells, activated fibroblasts, basement membranes, capillaries and the extracellular matrix (8). Recent studies have characterized different subsets of immune and stromal cells in the TME of PAs, as well as cytokines, growth factors and stromal remodeling enzymes (9). The basement membrane (BM) is a key element of the TME and is widely distributed in the extracellular matrix of metazoan tissues (10). The function of the BM is to resist mechanical stress, determine tissue shape and create diffusion barriers (11). The BM can also provide signals that guide cell polarity, differentiation, migration and survival (12). Because the mechanistic actions of the BM can affect morphological changes in tissues, it follows that the BM can also affect the proliferation, invasion and metastasis of tumor cells (13). The structure and properties of the BM are encoded and regulated by a specific suite of BM genes; variations in the expression of BM genes are considered to be the basis of many human diseases. In addition, proteins in the BM are the selective targets of autoantibodies in immune diseases (14, 15). Therefore, the combined study of BM genes and immune infiltration in IPAs may provide new clues for the clinical prevention, diagnosis, and treatment of this disease.

A recent study developed and defined a comprehensive network of more than 200 genes and proteins in the BM (16). On this basis, we used several bioinformatics methods to investigate the mechanisms underlying the specific action of BM genes in IPAs. Four datasets containing IPA and NIPA samples were downloaded from the Gene Expression Omnibus database (GEO) to obtain expression data from genes in the BM. Then, we developed a protein-protein interaction network (PPI) that specifically related to genes in the BM. Next, we analyzed the correlation between the expression of BM genes and immune infiltration to develop a risk model to predict the invasiveness of PAs. Finally, we validated our findings by performing qPCR on samples acquired from surgery and recommended 10 possible drugs.

## Materials and methods

### Data acquisition and processing

**Figure 1:**
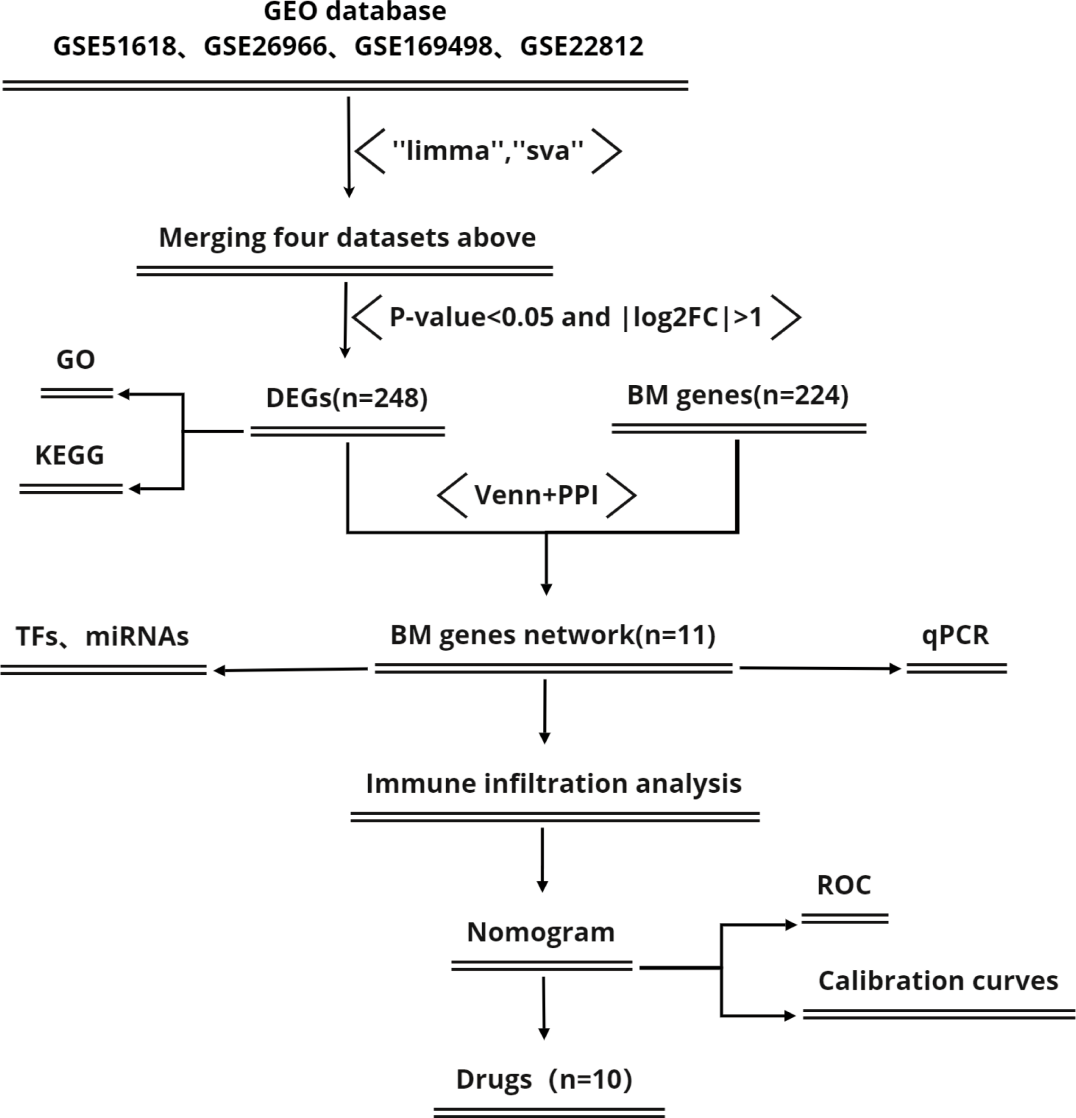
Flowchart showing the study process.

**Figure 1** shows a flowchart of the study process. First, we downloaded four IPA- related datasets (**Table 1**) from the Gene Expression Omnibus (GEO) database of the National Center for Biotechnology Information (NCBI): GSE51618, GSE26966, GSE169498, and GSE22812. These datasets were then merged with the “limma” package in R software (version 4.2.1); the “sva” package was used to remove lot-to-batch differences and other unwanted variations (17).

**Table 1:** Information relating to the four microarray datasets.

Accession numbers	Platform	Non-invasive PA	Invasive PA	Species
GSE51618	GPL6480	4	3	*Homo sapiens*
GSE26966	GPL570	7	7	*Homo sapiens*
GSE169498	GPL22120	24	49	*Homo sapiens*
GSE22812	GPL2895	5	8	*Homo sapiens*

### Identification of differentially expressed genes

Differentially expressed genes (DEGs) were identified by comparing expression levels between IPA samples and NIPA samples in the merged dataset using the “limma” package (18); the screening criteria were a *P* < 0.05 and |log2FC| > 1. “Heatmap” and “ggplot2” packages were used to create heatmaps and volcano plots to visualize DEGs (19).

### DEGs enrichment analysis

To investigate the enrichment pathways and functions of DEGs, we performed the gene ontology (GO) and Kyoto Encyclopedia of Genes and Genomes (KEGG) enrichment analyses to explore biological significance and key approaches. For this, we used the “cluster Profiler”, “ggplot2”, “org.Hs.eg.db” and “enrichplot” packages of R software; *P* < 0.05 was considered as significant enrichment (20, 21).

### Acquisition of BM genes and construction of a PPI network

We identified genes in the BM with differential expression by identifying intersecting DEGs. Next, we used the STRING database (https://string-db.org) to construct a PPI network (with a confidence score of 0.4) for the identified genes; irrelevant genes were removed according to the degree of the connection and a cluster composed of interconnecting BM genes was obtained for subsequent analyses and risk model construction.

### Recognition of transcription factors and miRNAs

Transcription factors (TFs) are proteins that attach to specific genes and control the rate of DNA transcription. TFs recognize specific DNA sequences to control chromatin and transcription and form a complex system that directs genomic expression and underlies many different aspects of human physiology, disease, and variation (22). Topologically trusted TFs that often bind to genes were identified using the JASPAR database in NetworkAnalyst (23), an online platform for analyzing gene expression data and gaining insight into biological mechanisms, roles, and interpretations. In addition, miRNAs can be used to track target gene interactions that result in binding to gene transcripts that are detrimental to protein expression; in the present study, we used Tarbase as an experimental validity database of miRNAs-genes interactions (24, 25). Using this database, we extracted miRNAs interacting with genes in the PPI network and performed topological analysis to explore their biological functions and characteristics.

### Analysis of immune infiltration

Next, we used several packages in R (“ggpubr”, “GSVA”, “GSEABase” and “reshape2”) to analyze immune infiltration and correlations in samples and to analyze differences in the infiltration of immune cells and immune functions between IPA samples and NIPA samples. These results were presented as heatmaps and correlation matrix maps.

### Correlation analysis of BM genes and immunity

The ssGSEA algorithm was used to quantify the relative infiltration levels of immune cells and immune functions in the merged dataset (26). Next, we performed Spearman’s correlation analysis of immune cells and determined their relative functions in the BM gene cluster. Then, “psych” and “ggcorrplot” packages in R were used to visualize rectangular correlation plots.

### Risk model construction and verification

A risk prediction model for IPA was constructed by considering the correlations between the expression of BM genes and immune infiltration; this model was visualized by a nomogram. A calibration curve and receiver operating characteristic (ROC) curve were then used to evaluate and validate the model.

### Sample selection and qPCR validation

To validate our bioinformatics results, we used qPCR to verify the expression levels of the top three genes contributing to the risk model (*THBS2, SDC4* and *LUM).* The samples of PA tissues collected in this study were acquired from PA patients who underwent endoscopic transsphenoidal pituitary surgery in the Neurosurgery Department of The First Affiliated Hospital of Xiamen University between December 2021 and August 2022. All tissue samples were frozen in liquid nitrogen immediately after surgical resection and stored in a -80°C refrigerator to await further analysis; five IPA tissue samples and five NIPA tissue samples were acquired. All of the selected samples were confirmed by preoperative imaging, intraoperative endoscopic observation and postoperative pathology. IPAs were defined according to Knosp classification grades III-IV (27). All participants and their families provided signed and informed consent and the study was approved by the ethics committee of hospital.

Total RNA was extracted from samples using TRIzol reagent (Servicebio, Wuhan, China) for reverse transcription and SYBR Green qPCR Master Mix (None ROX) for qPCR in accordance with the manufacturer’s instructions. We used the *GAPDH* gene as an internal reference and the primer sequences are summarized in **Supplementary Table S1**. Each sample was repeated at least three times. The relative expression levels of *THBS2*, *SDC4* and *LUM* were calculated using the 2^-△△CT^ method. Differences in expression were analyzed by GraphPad prism (version 8.0.1) and the student’s t-test was used to determine the significance of the differences between groups. *P* < 0.05 was considered statistically significant.

### Drug recommendation

The DSigDB is a global database for identifying gene-related targeted drugs (28) and was used as a drug signature database to identify drug molecules. Potential drugs were identified by using the cluster of BM genes in the risk prediction model and the ‘diseases/drugs’ function in the online web tool Enrichr (https://maayanlab.cloud/Enrichr/ ).

### Statistical analysis

All data processing and analysis were performed in R software (version 4.2.1) and GraphPad prism (version 8.0.1). To compare continuous variables between the two groups, the statistical significance of normally distributed variables was calculated by the independent student’s t-test. Differences between non-normally distributed variables were calculated by the Wilcoxon rank-sum test. Chi-squared tests or Fisher’s exact test were performed to determine statistical significance of categorical variables between the two groups. Correlation coefficients between different genes were determined by Pearson correlation analysis. All statistical p-values were two-sided and *P* < 0.05 was considered statistically significant.

## Results

### Data processing and the identification of DEGs

**Figure 2:**
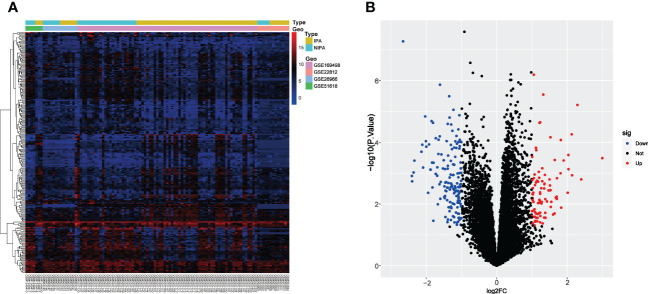
The identification of DEGs between IPA and NIPA samples in the merged dataset. **(A)** Heatmap, red rectangles represent high expression while blue rectangles represent low expression. **(B)** Volcano plot, red dots represent up-regulated genes, blue dots represent down-regulated genes, and black dots represent genes with no significant difference.

After removing the batch effect and merging the four datasets, 248 DEGs were identified between the IPA and NIPA samples after screening the merged dataset using *P* < 0.05 and |log2FC| > 1 as criteria. This included 91 up-regulated genes and 157 down-regulated genes; these were visualized as a heatmap (**Figure 2A**) and a volcano plot (**Figure 2B**).

### DEG enrichment analysis

**Figure 3:**
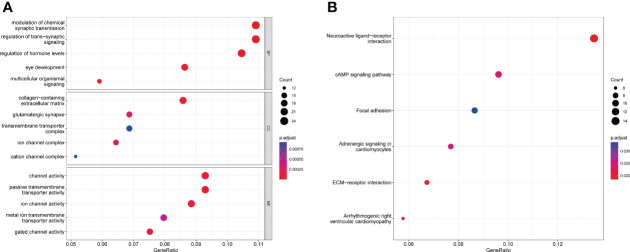
DEG enrichment results. **(A)** Bubble chart for GO analysis showing the top five GO terms for biological process (BP), cellular component (CC) and molecular function (MF). **(B)** Bubble chart for KEGG pathway analysis showing the top six enriched KEGG pathways in DEGs.

GO enrichment analysis showed that for biological processes, the DEGs were significantly enriched in the modulation of chemical synaptic transmission, the regulation of transsynaptic signaling and the regulation of hormone levels. For cellular components, DEGs were enriched in the collagen-containing extracellular matrix, glutamatergic synapses and the transmembrane transporter complex. For molecular functions, the DEGs were enriched in channel activity, passive transmembrane transporter activity and ion channel activity (**Figure 3A**). KEGG enrichment analysis showed that DEGs were mainly enriched in neuroactive ligand-receptor interaction, cAMP signaling pathways, ECM-receptor interaction and other related pathways (**Figure 3B**). Further details are provided in **Supplementary Table S2, S3.**

### Identification of BM genes and construction of a PPI network

**Figure 4:**
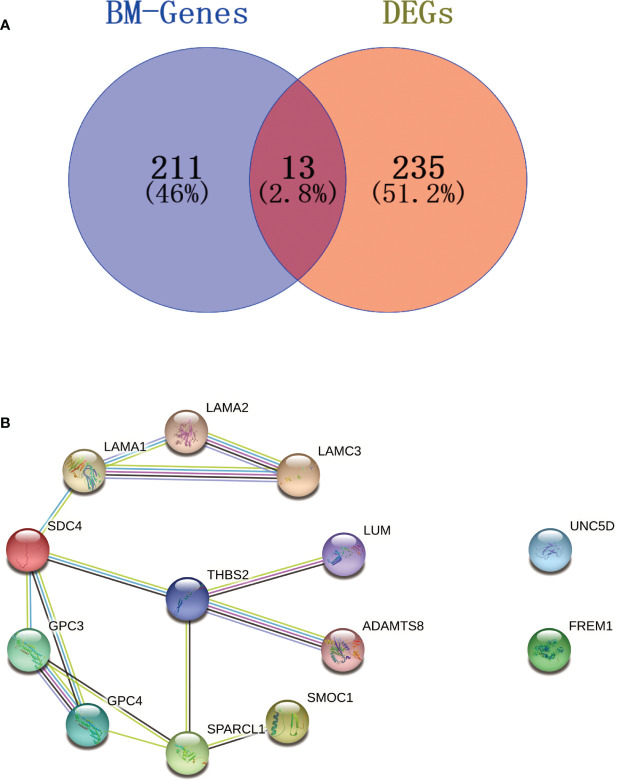
Venn diagram and PPI network. **(A)** Venn diagram of BM genes and DEGs; the intersection in the middle represents the identification of BM genes from DEGs. **(B)** PPI network; the cluster on the left shows how the 11 BM genes were connected with each other; the two genes on the right were irrelevant genes.

In total, 13 BM genes and their expression levels were extracted for the selected DEGs (**Figure 4A**). These were input into the STRING database to construct a PPI network with a confidence score of 0.4. A cluster of 11 BM genes was then obtained for subsequent analysis after removing irrelevant genes (*UNC5D* and *FREM1*): *SPARCL1, GPC3, LAMA1, SDC4, GPC4, ADAMTS8, LAMA2, LAMC3, SMOC1, LUM* and *THBS2* (**Figure 4B**). Further details of the 224 BM genes and 248 DEGs are provided in **Supplementary Table S4**.

### Identification of TFs and miRNAs

**Figure 5:**
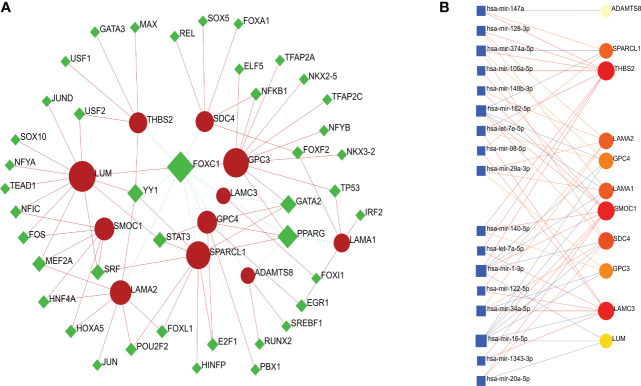
**(A)** A cohesive and regulatory BM genes–TFs interaction network; the rhombic nodes are TFs and interactions between gene symbols and TFs are shown as circle nodes. **(B)** An interconnected regulatory BM genes–miRNAs interaction network; the circle nodes indicate miRNAs and interactions between gene symbols and miRNAs are shown as squares.

To identify substantial changes occurring at the transcriptional level and gain insight into the molecules that regulate these 11 BM genes, we employed a network-based approach to decode regulatory TFs and miRNAs. Then, we used NeworkAnalyst to generate a BM genes-TFs interaction network (**Figure 5A**) and a BM genes-miRNAs interaction network (**Figure 5B**). It was evident that both TFs and miRNAs were closely related to the 11 BM genes, thus indicating that their characteristic features were regulated by more than one BM gene; furthermore, there was evidence of strong levels of interaction between the 11 BM genes.

### Analysis of immune infiltration

**Figure 6:**
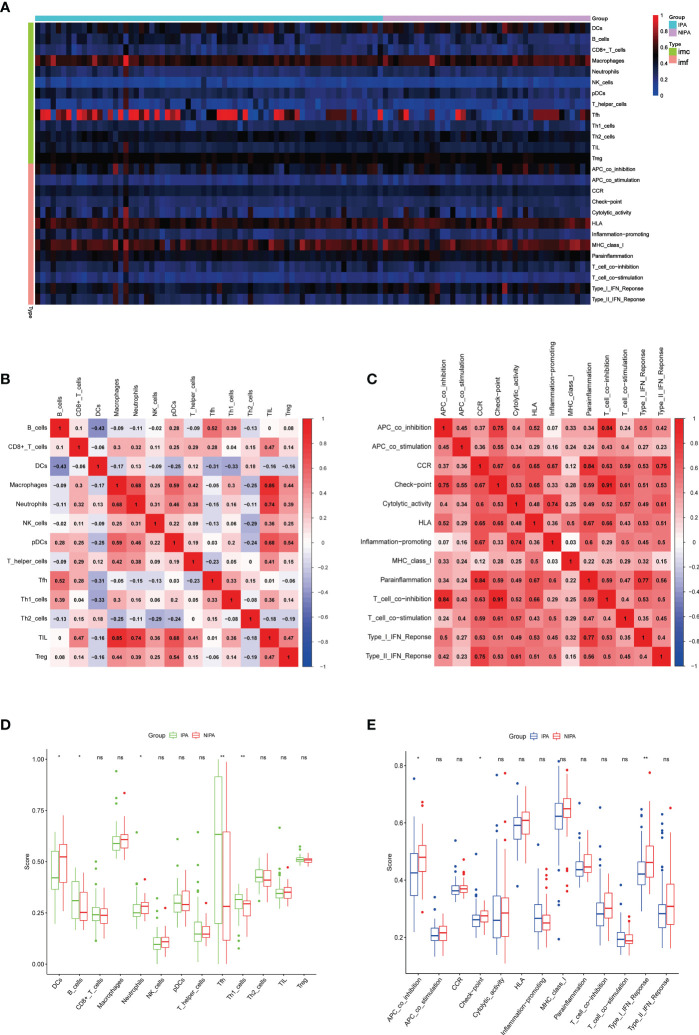
Analysis of immune infiltration. **(A)** Immune-related heatmap. **(B)** Rectangular diagram of immune cells. **(C)** Rectangular diagram of immune function. **(D)** Differences of immune cells when compared between IPA samples and NIPA samples. **(E)** Differences in immune function when compared between IPA samples and NIPA samples. * indicates *P* < 0.05, * indicates *P* < 0.01, ns indicates no significance.

Immune infiltration analysis showed that immune cells and immune functions differed with respect to their correlations (**Figure 6A**). Immune cells showed different degrees of positive and negative correlation (**Figure 6B**); the most significant correlation was between tumor infiltrating lymphocytes (TILs) and macrophages for which immune function consistently showed positive correlation (**Figure 6C**). Furthermore, several types of immune cells, including DCs, B cells, Neutrophils, Tfh and Th1 cells (**Figure 6D**) and immune functions such as APC co-inhibition, check point, and type I IFN response (**Figure 6E**) differed significantly between the IPA and NIPA samples.

### Correlation analysis of BM genes and immunity

**Figure 7:**
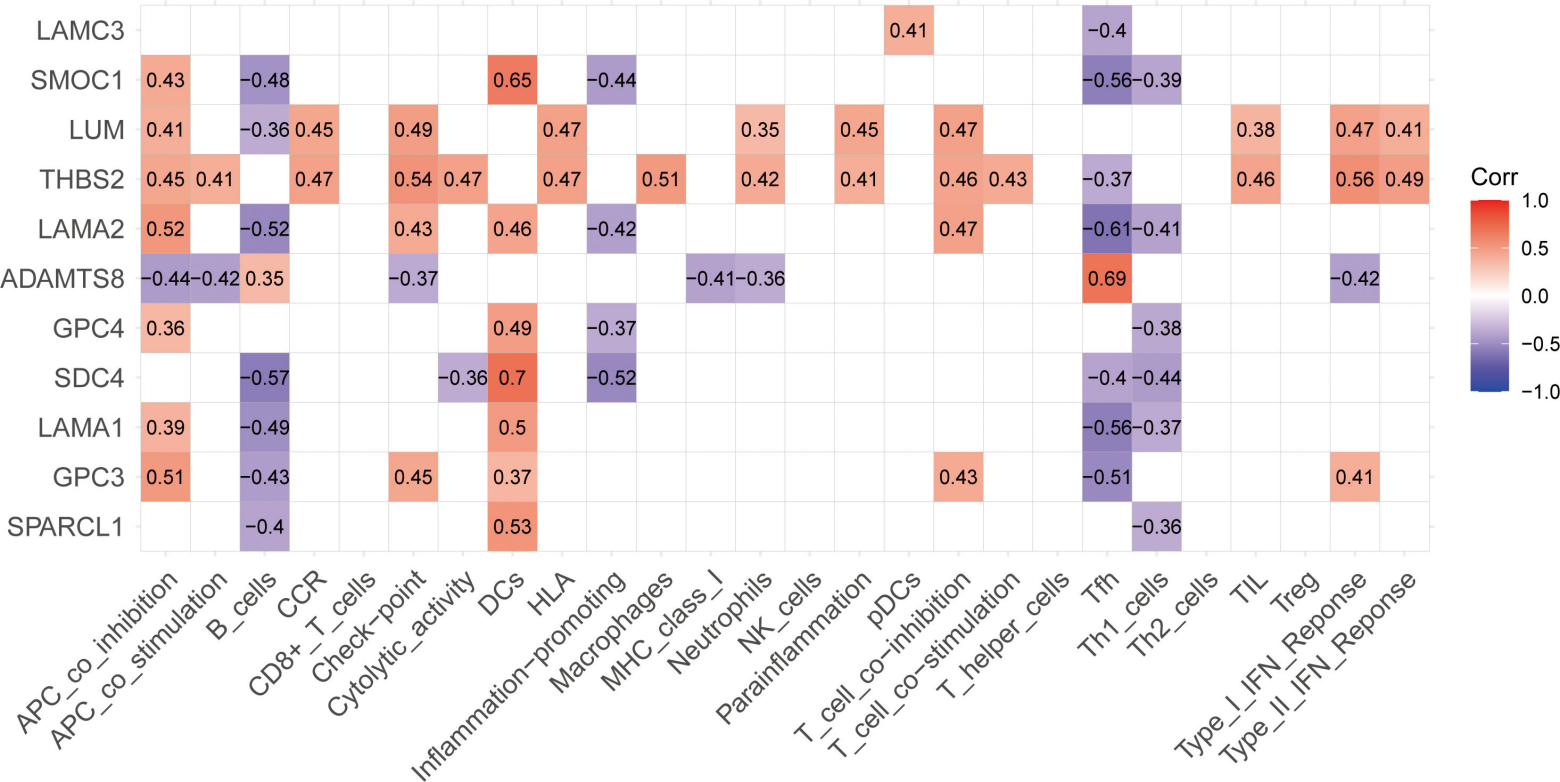
Correlation rectangle diagram for 11 BM genes and immunity showing correlations between the 11 BM genes and immunity including immune cells and immune functions; red indicates a positive correlation, purple indicates a negative correlation and the number in the box is the correlation coefficient.

According to the analysis of the expression levels of 11 BM genes and immune infiltration, we constructed a correlation rectangle diagram (**Figure 7**) which showed that the 11 BM genes identified had different degrees of correlation with immune cells and immune function. The most significant positive correlation was between *SDC4* and DCs. *LAMA2* exhibited the most significant negative correlation with Tfh (r=-0.61). *THBS2* was correlated with the highest number of immune cells and immune function items; all were positively correlated except for Tfh.

### Construction and verification of a risk prediction model

**Figure 8:**
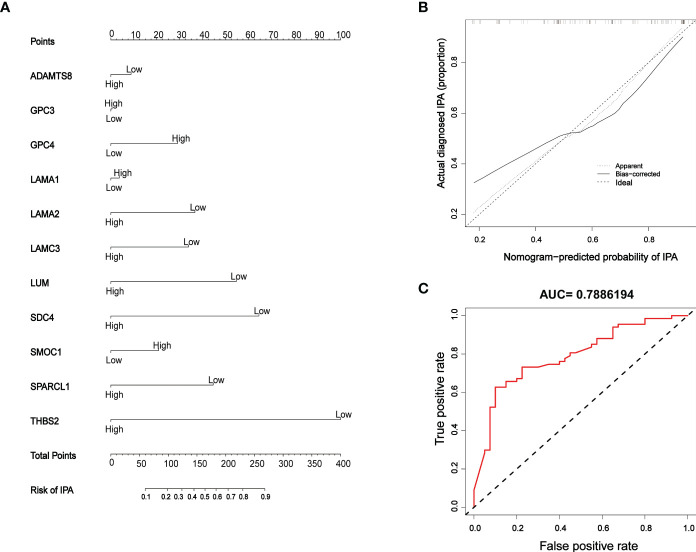
Model establishment and verification **(A)** Nomogram; ‘high’ and ‘low’ represent gene expression levels. **(B)** Calibration curves; the dashed diagonal line in grey represents the ideal prediction by a perfect model. The closer the bias-corrected calibration curve (red line) is to the diagonal line, the higher the prediction accuracy of the model. **(C)** ROC curve; the area under the red curve represents the AUC.

Next, a risk model for IPAs was constructed based on the results arising from complex correlation between the 11 BM genes and immunity; the model was presented as a nomogram (**Figure 8A**) which was able to generate individual probabilities of clinical events by integrating different variables, thus meeting the need for biological and integrated models and achieving the promotion of personalized medicine (29). All 11 genes contributed to the model although *THBS2* contributed the most and accounted for the highest score. Calibration curves were used to visualize the performance of the nomogram, thus confirming the performance of the evaluation model (**Figure 8B**). The effectiveness of the model to predict the invasiveness of PAs was verified by ROC analysis (AUC: 0.7886194), thus proving that the model exhibited good evaluation value (**Figure 8C**).

### qPCR validation

**Figure 9:**
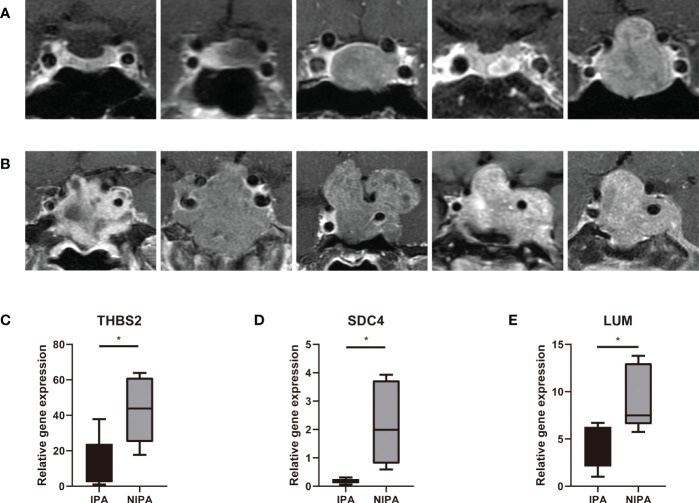
qPCR detection. **(A)** Preoperative MRI scans from five patients with NIPAs. **(B)** Preoperative MRI scans from five patients with IPAs. **(C–E)** Expression levels of *THBS2*, *SDC4* and *LUM* in IPA and NIPA tissues along with statistical results arising from unpaired t-tests, * indicates *P* < 0.05.

The ten clinical samples used for qPCR were obtained from five patients with NIPAs (**Figure 9A**) and five patients with IPAs (**Figure 9B**); analysis showed that the expression levels of *THBS2* (*P* = 0.0196), *SDC4* (*P* = 0.016) and *LUM* (*P* = 0.0284) in IPA tissues were significantly lower than those in NIPA tissues (**Figure 9C–E**). These findings were consistent with those arising from DEGs analysis, thus indicating that the data mining was reliable and the model has potential research value.

### Drug recommendations

Target-drug interaction analysis can reveal interactions between drugs and targets that are critical if we are to understand the structural features necessary for receptor sensitivity (30). The 11 BM genes featuring in the model were used as drug targets to extract the top 10 drugs from the DSigDB database according to *P*-value. Then, we obtained the chemical and structural formulae for each drug from the Drugbank database (**Table 2**).

**Table 2:** The top 10 recommended drugs.

Name	P-value	Chemical Formula	Structure
Dasatinib CTD 00004330	0.00224428	C22H26ClN7O2S	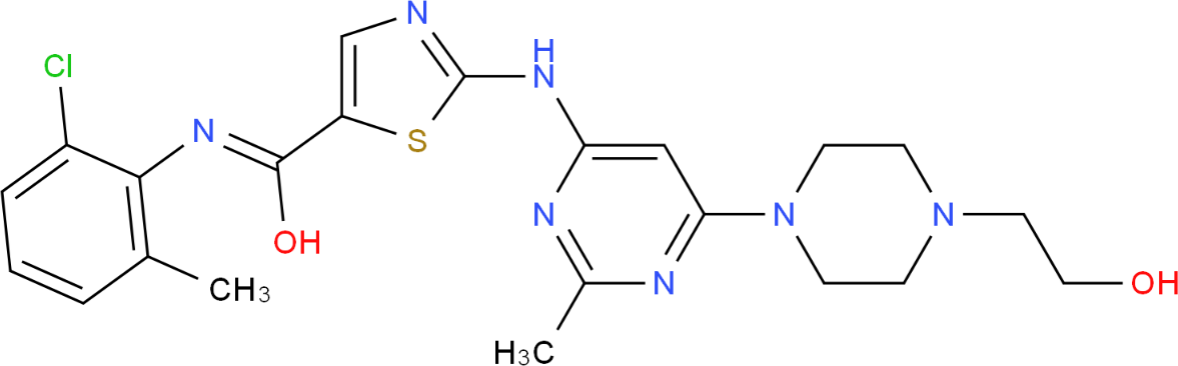
Progesterone CTD 00006624	0.002262266	C21H30O2	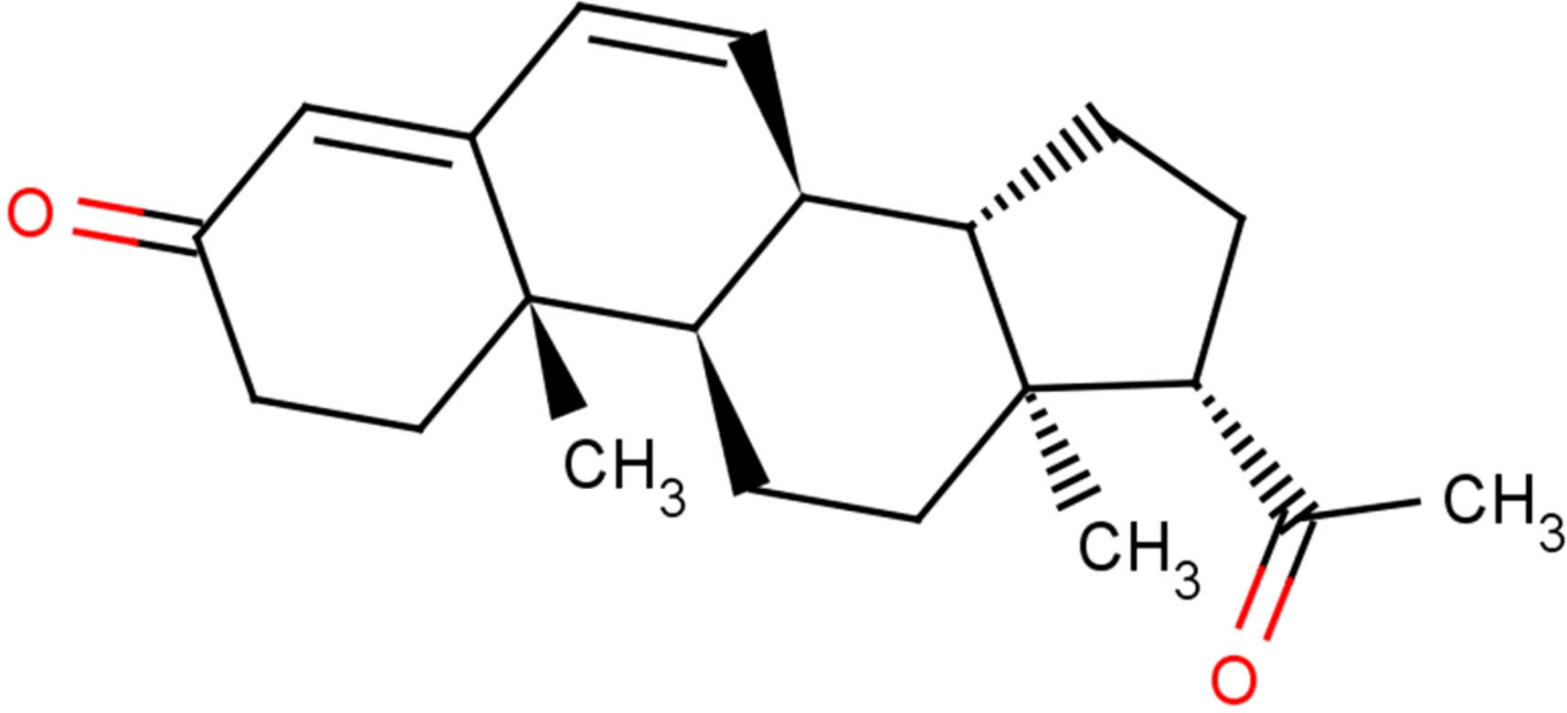
Retinoic acid CTD 00006918	0.002879104	C20H28O2	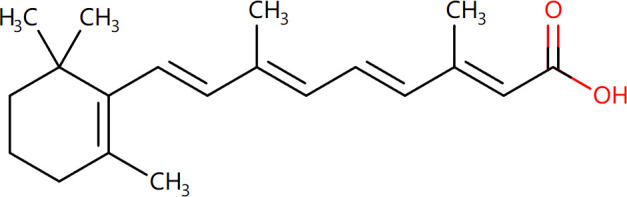
Trichostatin A CTD 00000660	0.006687154	C17H22N2O3	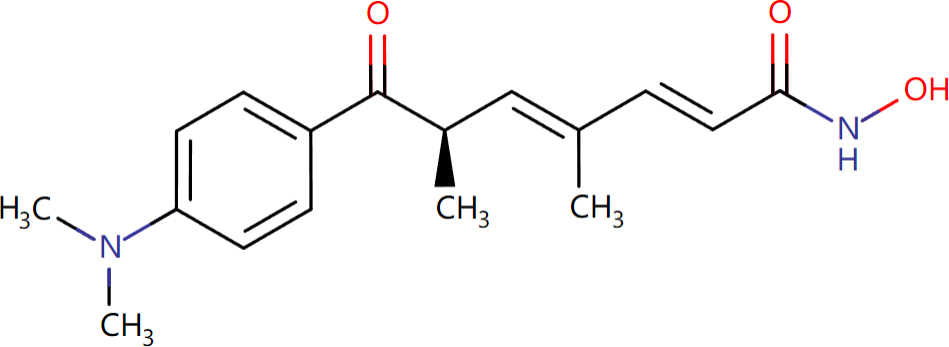
Arbutin CTD 00005438	0.010086441	C12H16O7	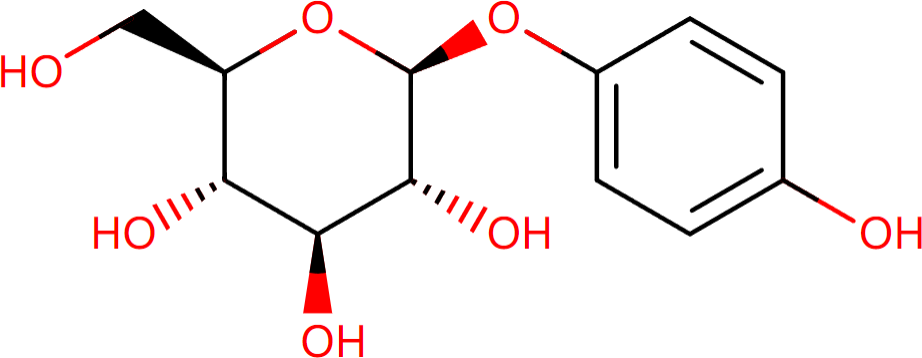
Decitabine CTD 00000750	0.012900927	C8H12N4O4	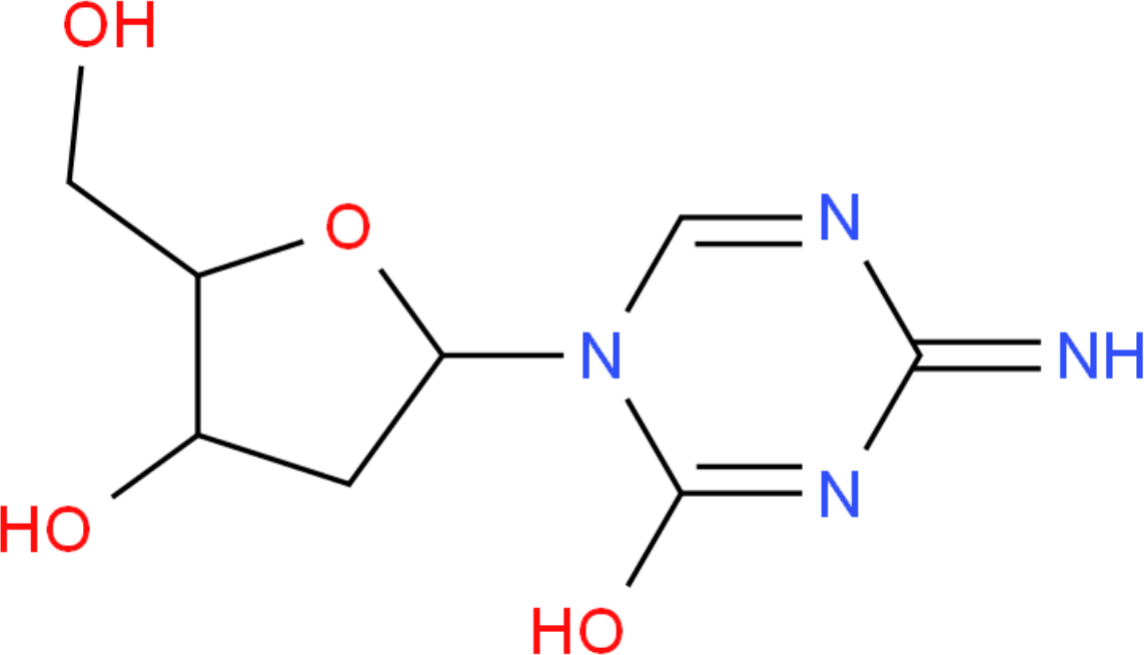
Mifepristone CTD 00007083	0.013922574	C29H35NO2	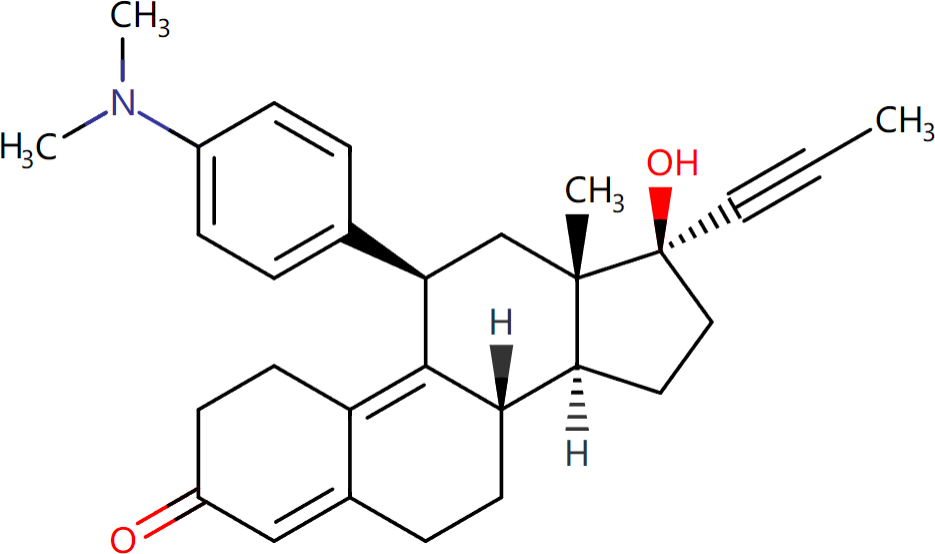
Furazolidone HL60 DOWN	0.020167647	C8H7N3O5	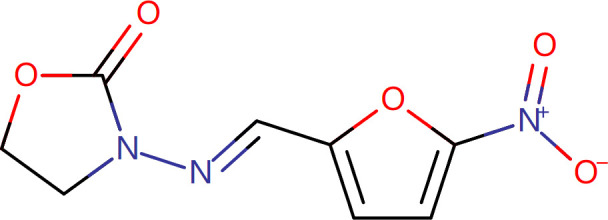
Latamoxef HL60 UP	0.024508037	C20H20N6O9S	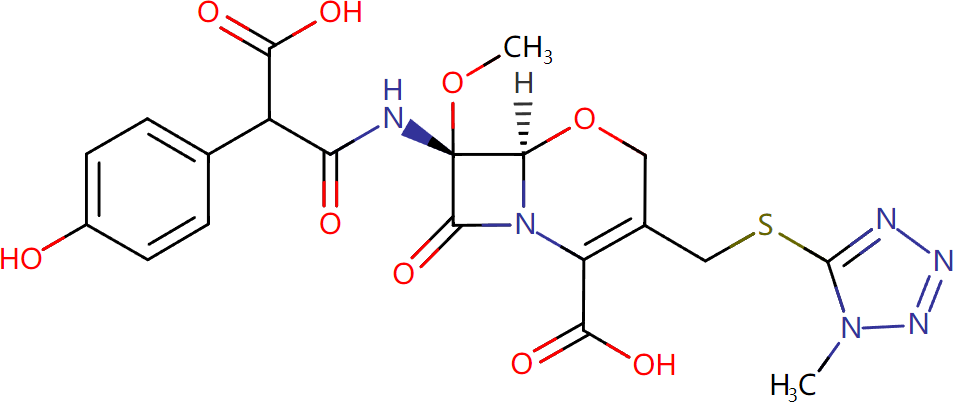
Cyclosporin A CTD 00007121	0.029019386	C62H111N11O12	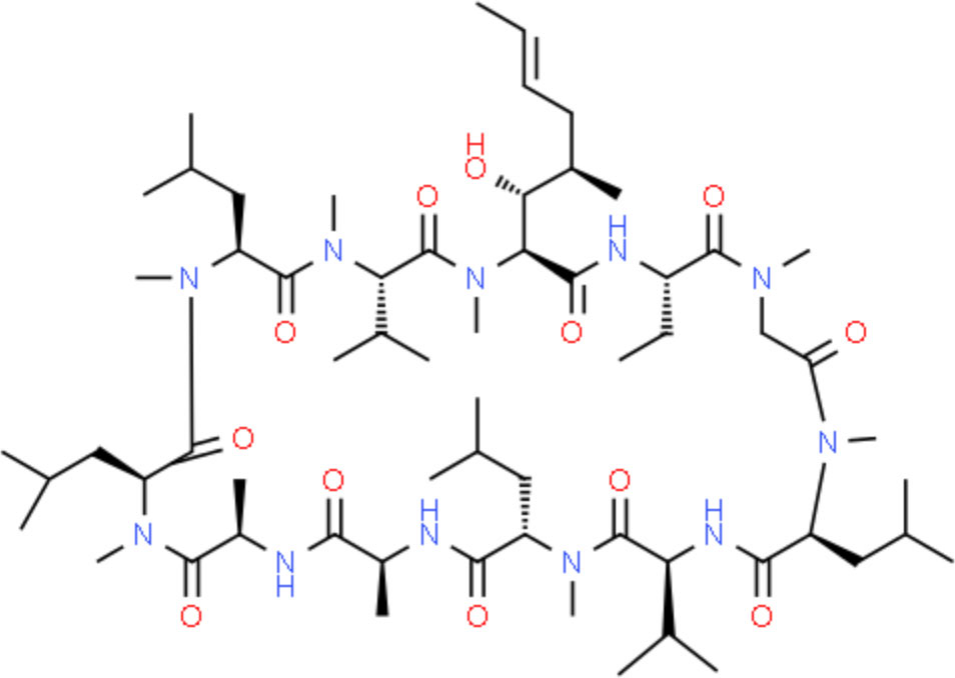

## Discussion

IPAs are common and intractable intracranial epithelial-derived tumors with multiple causes, processes and outcomes (31). Recent advances in the field of molecular medicine have shown that molecular changes at the levels of the genome, transcriptome, proteome, and metabolome are all involved in the potential invasion of PAs (32). In addition, the altered expression of some genes and the presence of mutations are also associated with the invasiveness of PAs (33). However, the mechanism underlying invasion and proliferation have yet to be fully elucidated. The BM is a special type of extracellular matrix that is present in epithelial-derived tumors. Tumor cells must repeatedly destroy and overcome this barrier to invade adjacent structures or metastasize (34, 35). Once integrity is lost, forces from overlying differentiated tumor cells may mechanically drive the invasion of tumor progenitor cells at stromal boundaries (36). Previous studies have shown that the TME is essential for tumor growth, invasion and metastasis (37) and the interaction between tumor cells and associated stroma also represents a powerful relationship that might affect disease initiation, progression and prognosis (38). As an important component of the TME and an indispensable extracellular matrix of PAs, the BM not only affects immune infiltration, but also affects tumor invasiveness. Since the structure and properties of the BM are encoded and regulated by specific genes expressed in the BM, we specifically investigated the mechanistic action of BM genes in IPAs.

Four datasets, featuring IPAs and NIPAs, were downloaded from the GEO database, merged, and then screened for DEGs. Enrichment analysis of the DEGs suggested that GO terms were mainly enriched in signal transduction, extracellular matrix, and channel activity. KEGG analysis revealed enrichment in neuroactive ligand-receptor interaction, the cAMP signaling pathway and ECM-receptor interaction. Signal transduction and channel activity play an important role in the endocrine function of the pituitary gland (39). As a key element of the TME, ECM degradation affects the proliferation, invasion and metastasis of tumor cells (40). These pathways may be involved in the disease progression of IPAs under the regulation of the DEGs identified.

In order to further investigate the roles and mechanistic actions of BM genes in IPAs, we extracted 13 BM genes from the DEGs. After removing the irrelevant genes by PPI network analysis, a cluster composed of 11 closely connected BM genes was obtained, including *SPARCL1, GPC3, LAMA1, SDC4, GPC4, ADAMTS8, LAMA2, LAMC3, SMOC1, LUM* and *THBS2*. Most of the genes were expressed at low levels and may contribute to the reduction or degradation of BM stiffness to promote tumor invasion; similar results were reported previously (36). Next, we used the 11 BM genes to generate a BM genes-TFs interaction network and a BM genes-miRNAs interaction network to identify transcriptional and post-transcriptional regulators. Of the identified TFs, FOXC1 is known to inhibit the migration and invasion of pituitary tumor cells under the regulation of has-mir-133; furthermore, members of the FOXC1 family are already known as drug targets for cancer (41, 42). PPARG has been found to directly regulate pituitary function in mice (43). YY1, as a transcriptional repressor, has been found to be related to the sequence of hypersensitive site V in the control region of the human growth hormone gene locus (44). The expression of GATA2 in PAs can be detected by immunochemistry and used to identify gonadotropic PAs; furthermore, the interaction between GATA2 and Pit-1 can lead to gene-specific action and the differentiation of TSH-secreting PAs (45, 46). Previous studies have shown that miRNA markers are promising biomarkers for the treatment of different types of PAs (47). For example, has-mir-34c-3p, has-mir-34B-5p, has-mir-378 and has-mir-338-5p were all significantly down-regulated in in prolactin PAs, while the down-regulation of has-mir-493-5p and the up-regulation of has-mir-181b-5p has been detected in NFPAs (48). Furthermore, the expression of has-mir-184 was found to be significantly up-regulated while the expression of has-mir-124-3p was down-regulated in GH-secreting PAs (48). Furthermore, has-mir-143 has been shown to inhibit cell proliferation and promote cell apoptosis by targeting K-Ras (49). In a previous study, Vicchio et al. found that the downregulation of has-mir-26b-5p and has-mir-30a-5p was negatively associated with ki-67, atypical morphological features, and invasion of the cavernous sinus (50). Of these interrelated miRNAs, hsa-mir-128-3p, hsa-let-7e-5p, hsa-mir-98-5p, hsa-mir-29a-3p, hsa-mir-140-5p, hsa-mir-34a-5p, and hsa-mir-20a-5p have all been related to the occurrence and development of tumors (51). Investigating the underlying mechanisms of TFs and miRNAs may be important for understanding the invasiveness of IPAs and may help us to discover new potential biomarkers and yield innovative therapies.

Next, we compared immune infiltration between IPA tissues and NIPA tissues. We identified significant differences between the two groups with regards to DCs, B cells, neutrophils, Th1 cells and Thf cells. DCs have been shown to be present in the pituitary gland and play an important role in immune activation of the hypothalamic-pituitary-adrenal (HPA) axis; the maturation and migration of DCs to lymphatic tissue is key to developing an immune response or maintaining tolerance (52). B cells have been shown to exhibit higher levels of infiltration in aggressive GH-secreting PAs; this was related to overactivation of the JAK-STAT pathway (53). In addition, the expression of IL-10 by tumor cells and macrophages has been shown to promote the survival of B cells and lymphomas by producing TNF family member B cell activators and IL-10, thus suppressing the adaptive immune response; this mechanism is thought to facilitate the evasion of immune surveillance (54). Neutrophils, Th1 cells and Thf cells are important immune cells in the TME. Research has shown that neutrophils may determine tumor proliferation and angiogenesis while the polarization of immune response in Th1 cells can stimulate anti-tumor immunity and inhibit the progression of PAs. The IL-21 produced by Thf cells has been shown to be necessary for the inhibition of tumor and CD8 cell function (55–57). In the present study, the immune cells and immune functions in the two groups of samples were found to be interrelated; the strongest correlation among immune cells was between macrophages and TILs. The lactic acid secreted by IPAs can promote M2 polarization in macrophages, thus contributing to angiogenesis and the inhibition of immune response; this process can also cause the release of CCL17 to enhance the invasion of PAs *via* the CCL17/CCR4/mTORC1 axis (58, 59). TILs were found to be positively related to macrophages and is currently being used as an emerging immunotherapy for solid tumors; in addition, TILs have been successfully applied for the treatment of some tumors (60). These results are consistent with the positive correlation between various co-inhibition and co-stimulation in the correlation analysis of immune function in the samples. Moreover, we identified many important immune correlations; these correlations and targets may provide new concepts for the treatment of IPAs. On this basis, we performed correlation analysis between the 11 BM genes and immunity and found that the 11 genes were all correlated with immunity to differing degrees; *THBS2, LUM, SDC4, SMOC1* and *ADAMTS8* exhibited the most immune-related items or higher correlation coefficients. *THBS2* had the largest number of association items; most of these were positively correlated. Moreover, the value of *THBS2* in tumor immunity and diagnosis has been confirmed in several previous studies (61–64); however, the value of *THBS2* in IPAs has not been reported previously. LUM, a member of the small leucine-rich proteoglycans (SLRP) family, is a component of the extracellular matrix (65). Low expression levels of *LUM* at the tumor margin in malignant melanoma may promote the proliferation of melanoma cells; however, whether *LUM* acts as a tumor suppressor or oncogenic gene depends on the cellular environment and thus related to the TME (66). Analysis showed that *SDC4* and *SMOC1* had similar immune-related performance and both exhibited strong positive correlations with DCs; other genes exhibited negative correlations. SDC4 is a transmembrane proteoglycan that binds to the ECM and soluble factors *via* extracellular glycosaminoglycan chains. In a previous study, Horiguchi et al. demonstrated that SDC4 mediated the formation of stress fibers in the anterior pituitary hair follicle cells of rats, thus resulting in key morphological changes (67). *SMOC1* was first discovered in 2002, is widely distributed in the basement membrane ECM of many tissues (68), and is known to be upregulated in oligodendrocytoma and astrocytic tumors and can inhibit the migration of glioma U87 cells induced by tenascin C (69). As a therapeutic target for T cell-associated immune responses, *ADAMTS8* was negatively correlated with most of the immune responses in the result. The only immune cell that was positively correlated with *ADAMTS8* was Tfh; this association helped to develop or support the recruitment sites of CD8^+^T cells, NK cells, and macrophages, while supporting the anti-tumor antibody response of B cells (70, 71). *LAMA2*, *GPC4* and *SPARCL1* have been found to be associated with NFPAs in recent studies; the expression and methylation status of *LAMA2* are known to be related to the invasiveness of NFPAs (72). Furthermore, a previous study identified significant differences in the expression levels of *GPC4* when compared between normal pituitary glands and invasive NFPAs (73). Thus, *SPARCL1* can be used as a potential diagnostic or prognostic marker in patients with NFPAs and also represents a potential therapeutic target (74). *LAMC3* (75), *LAMA1* (76) and *GPC3* (77) also play important roles in the progression of malignant tumors and immunotherapy.

Although associations between IPAs and genetic or clinical variables have been reported previously, it is rare to include multiple variables in the assessment of invasiveness. In this study, a risk model for IPAs was established based on the immune infiltration results of 11 BM genes that exhibited complex associations with IPAs. The low expression levels of *THBS2, SDC4, LUM, SPARCL1, LAMA2, LAMC3*, and *ADAMTS8* could increase the risk of PA invasiveness, especially low expression levels of *THBS2*, *SDC4* and *LUM*, as verified by qPCR. Furthermore, the high expression levels of *GPC4, SMOC1, LAMA1* and *GPC3* could increase the risk of PAs invasiveness. Collectively, the expression levels of each gene in the model collectively correspond to the risk coefficient of IPAs. The predictive value of the model was evaluated by calibration curve and ROC analysis; these analyses proved that the accuracy of the model for predicting the invasiveness of PA was satisfactory (AUC=0.7886194), thus providing a reference for clinical diagnosis and individualized treatment planning. Finally, 10 possible drugs were recommended for the 11 BM genes included in the model, which may provide the possibility of drug treatment for IPAs and facilitate researchers interested in this field to conduct further research from the perspective of genes and drugs.

To the best of our knowledge, this is the first comprehensive study to predict the invasiveness of PAs based on the correlations between BM genes and immunity. Our findings may provide new possibilities for the prevention, diagnosis and treatment of IPAs. However, this study still has some limitations that need to be considered. Firstly, this study involved a comprehensive prediction model based on multiple types of PAs from multiple centers; thus, the model may show bias when predicting the invasiveness of a specific type of PA. Additional validation, incorporating more accurate and extensive clinical data is now needed to improve the accuracy of the model. Secondly, although qPCR showed that the expression levels of BM genes were consistent with the results of bioinformatics, the specific role and biological mechanisms underlying the action of BM genes in the invasiveness of PAs need to be investigated further. Furthermore, our findings, and the potential mechanisms involved in the correlations between the 11 BM genes and immune infiltration also need to be verified and explored by complex molecular biology experiments or flow cytometry analysis in future research.

## Conclusion

We used a variety of bioinformatics methods to develop a nomogram model to predict the invasiveness of PAs based on the correlations between 11 selected BM genes and immune infiltration. These findings may facilitate closer surveillance and early diagnosis to prevent or treat IPAs in patients. our findings may also improve the awareness of clinicians with regards to patients at high risk of IPAs.

## Data availability statement

The datasets presented in this study can be found in online repositories. The names of the repository/repositories and accession number(s) can be found in the article/**Supplementary Material.**

## Ethics statement

The studies involving human participants were reviewed and approved by The First Affiliated Hospital of Xiamen University. The patients/participants provided their written informed consent to participate in this study. Written informed consent was obtained from the individual(s) for the publication of any potentially identifiable images or data included in this article.

## Author contributions

Conception and design: ZC. Writing and review of the manuscript: ZC and HZ. Data analysis and interpretation: ZC and XS. Samples collection and processing: ZC, YK, HZ and XL. Experiment performance and analysis: JZ and FJ. All authors contributed to the article and approved the submitted version.
